# BABA-Primed Histone Modifications in Potato for Intergenerational Resistance to *Phytophthora infestans*

**DOI:** 10.3389/fpls.2018.01228

**Published:** 2018-08-29

**Authors:** Barbara Meller, Daniel Kuźnicki, Magdalena Arasimowicz-Jelonek, Joanna Deckert, Jolanta Floryszak-Wieczorek

**Affiliations:** ^1^Department of Plant Physiology, Poznań University of Life Sciences, Poznań, Poland; ^2^Department of Plant Ecophysiology, Faculty of Biology, Adam Mickiewicz University in Poznań, Poznań, Poland

**Keywords:** potato leaves, priming, late blight, histone modifications, intergenerational SAR

## Abstract

In this paper we analyzed β-aminobutyric acid (BABA)-primed epigenetic adjustment of potato cv. “Sarpo Mira” to *Phytophthora infestans*. The first stress-free generation of the potato genotype obtained from BABA-primed parent plants via tubers and seeds showed pronounced resistance to the pathogen, which was tuned with the transcriptional memory of SA-responsive genes. During the early priming phase before the triggering stress, we found robust bistable deposition of histone marks (H3K4me2 and H3K27me3) on the *NPR1* (Non-expressor of *PR* genes) and the *SNI1* gene (Suppressor of *NPR1*, Inducible), in which transcription antagonized silencing. Switchable chromatin states of these adverse systemic acquired resistance (SAR) regulators probably reprogrammed responsiveness of the *PR1* and *PR2* genes and contributed to stress imprinting. The elevated levels of heritable H3K4me2 tag in the absence of transcription on SA-dependent genes in BABA-primed (F_0_) and its vegetative and generative progeny (F_1_) before pathogen challenge provided evidence for the epigenetic mark for intergenerational memory in potato. Moreover, our study revealed that histone acetylation was not critical for maintaining BABA-primed defense information until the plants were triggered with the virulent pathogen when rapid and boosted *PR*s gene expression probably required histone acetyltransferase (HAT) activity both in F_0_ and F_1_ progeny.

## Introduction

Sometimes pathogen challenge or chemical treatment can trigger in plant a unique physiological state in which a plant is conditioned for the superactivation of defenses against new unfavorable conditions. In the primed state of defense the plant responds faster and activates more rapid defense responses when it is treated with a much more severe biotic stress. An important role of priming in systemic acquired resistance (SAR) has been supported by findings that primed cells are subject to chromatin modifications by epigenetic marks affecting plant defense (e.g., Conrath, 2011; Jaskiewicz et al., 2011; Mauch-Mani et al., 2017).

Both short- and long-lasting environmental experience of the parent can be imprinted and passed on to the next progenies as epigenetic memory in the form of sustained histone modifications and DNA methylation changes, linked to chromatin remodeling states that reprogram stress response gene expression. Specific histone modifications retained at altered levels after removal of the stimulus associated with persistent active or repressed chromatin states are proposed to act as heritable marks that manage re-establishment of the parental chromatin patterns on offspring chromosomes that might be kept through mitotic or meiotic cell divisions (Avramova, 2015).

Methylation of lysine residues on histone H3 and H4 by methyltransferases can positively or negatively affect gene transcription. Generally, methylation of H3K4me2/3 and H3K36me2/3 is associated with transcription-permissive chromatin (Xu et al., 2008; Zhang et al., 2009), while H3K27me3, H3K9me2, and H4 methylated especially at Lys-20 are found in transcription-repressive chromatin (Bernatavichute et al., 2008; Zhang, 2008; Dong and Weng, 2013). Histone methylation is mainly mediated by SET {for Suppressor of variegation [SU(VAR)3-9], Enhancer of zeste [E(z)], and Trithorax [Trx]} domain protein methyltransferase. However, the memory of the transcriptionally active/inactive chromatin status under stress responses is maintained by two classes of proteins, termed the Trithorax (TrxG) and the Polycomb (PcG) groups (Liu et al., 2010). Generally, it is accepted that Trithorax mediated H3K4me2/3 keeps genes responsive to stress in activation (e.g., Schuettengruber et al., 2011), and antagonizes PcG activity, which establishes H3K27me3 and H3K9me2 at the target genes (e.g., Köhler and Hennig, 2010; Audergon et al., 2015). Owing to their broad spectrum of preferred genes, evidence for the role of TrxG/PcG protein complexes in regulating defense priming responses currently remains incomplete (Derkacheva and Hennig, 2014; Kleinmanns and Schubert, 2014; Pu and Sung, 2015).

Apart from histone methylases described as “writers,” the chromatin structure may be regulated by demethylases “erasers.” The lysine-specific demethylase 1 (LSD1)-like proteins and a larger class of Jumonji C-domain (JmjC) proteins are engaged in the elimination of methyl groups from the methylated histones (Shi et al., 2004; Chen et al., 2011). The unstable equilibrium between histone methylation and demethylation is involved in many plant physiological (e.g., vernalization) or pathophysiological states (Köhler et al., 2012; Kim and Sung, 2014).

The histone methylation process is a major determinant of chromatin conformation, although histone acetylation/deacetylation also plays an important role in gene transcription. There are many histone-acetyltransferases (HATs) and -deacetylases (HDACs) with differing preferences for mutual cooperation. It is considered that acetylated histones are associated with active transcription, whereas the hypoacetylated histones are involved in gene repression (Boycheva et al., 2014). Especially in view of intergenerational or transgenerational memory, an epigenetic mark, in contrast to chromatin mark, remains at an altered level after removal of the stress stimulus but rather influences the future transcriptional activity of the training gene for enhanced stress resistance (Avramova, 2015; Lämke and Bäurle, 2017).

Generally it has been known that β-aminobutyric acid (BABA), a non-protein amino acid, is an effective chemical agent in long-term metabolic and epigenetic memory improving plant resistance to biotic stresses (Slaughter et al., 2012; Worrall et al., 2012; Luna et al., 2014; Floryszak-Wieczorek et al., 2015; Martínez-Aguilar et al., 2016; Wilkinson et al., 2018).

As it was experimentally documented in *Arabidopsis*, transgenerational resistance induced by BABA was lost in the second generation in the absence of a new BABA treatment (Slaughter et al., 2012), while transgenerational resistance induced by *Pseudomonas syringae* DC 3000 lasted over one stress-free generation (Luna et al., 2012). Moreover, the long-lasting protection against the pathogen required the activity of the central immune regulator NPR1 (Non-expressor of *PR* genes) and was associated with SA-responsive genes (Luna et al., 2012). In *Arabidopsis thaliana* the transcription factor WRKY70, while in potato WRKY1, are needed for full expression of SA-responsive *PR-1*, *PR-2*, and *PR-5* genes (Li et al., 2004; Pieterse and Van Loon, 2004; Saubeau et al., 2016).

The activity of the SNI1 protein (Suppressor of NPR1, Inducible 1), a negative regulator of SAR required to dampen the expression of *PR* genes, is also controlled by the epigenetic machinery. As it was shown by Mosher et al. (2006), more than 90% genes up-regulated in *sni1* were benzothiadiazole S-methylester (BTH)-responsive and NPR1-dependent in *Arabidopsis*. Moreover, the study revealed that SNI1 inhibited SA-dependent transcription of the *PR-1* gene through repression of histone H3 acetylation and methylation of histone H3K4me2, while the *sni1* mutant contained high levels of histone H3 acetylation and H3K4me2 in the *PR-1* promoter region.

A key to the understanding of the epigenetic background and duration of priming is provided by the ability to search for molecular mediators of the post-stress information. The research of Jaskiewicz et al. (2011) provided evidence that local inoculation of *Arabidopsis* leaves with *Psm* (*P. syringae* pv. maculicola) or BTH application modified the status of methylation (H3K4me2/3) and acetylation (H3K9ac) at the promoter sequence of selected *WRKY* genes, which might create long-lasting post-stress memory in systemic leaves. In turn, López et al. (2011) based on *Arabidopsis* mutants blocked in RNA-directed DNA methylation revealed that primed responses are combined with post-translational histone modification, mainly by tri-methylation (H3K4me3) and acetylation (H3K9ac) at the promoter of the *PR1* gene.

Then, Luna et al. (2012) documented the effect of transgenerational priming in *Arabidopsis* conferred by the maintenance of a constant pressure of the virulent isolate of *P. syringae*, provided by the five times repeated inoculation in the parental generation. As a consequence, the first generation exhibited a primed state associated with the hypomethylated DNA status and chromatin modifications at the promoter of the *PR1*, *WRKY6*, and *WRKY53* gene.

Other studies have reported that NPR1-independent resistance of *Arabidopsis* to *H. arabidopsis* was transient and might be reset within 2 weeks, in contrast to the NPR1-dependent long-lasting BABA-primed epigenetic regulation managed by methyltransferase activity of H3K9 (Luna et al., 2014).

In the presented paper our intention was to analyze BABA-primed epigenetic marks with the potential to create and harbor an intergenerational memory of potato resistance to virulent *Phytophthora infestans*. In this study potato (*Solanum tuberosum* L.) was used, a non-model, but very important crop plant every year being the most devastated by *P. infestans* (Mont.) de Bary as the causative agent of late blight disease. We selected stress-responsive genes (*NPR1*, *SNI1*, *WRKY1*, *PR1*, and *PR2*) highly induced in response to *P. infestans*, by analyzing among others the chromatin-modifying activities of the Trx/H3K4me2 and SUVH4/H3K27me3 link with the transcriptional memory of these genes. To the best of our knowledge so far there have been no published data on the epigenetic control for potato intergenerational resistance to *P. infestans*.

Until now, research has mainly focused on searching for the presence of epigenetic marks in the promoter region; however, we adopted the chromatin immunoprecipitation (ChIP) protocol to look at the gene body sequence of the stress-responsive genes. Genetic evidence revealed that in plants, in contrast to animals, H3 methylation marks (e.g., H3K4me1-3) are mainly located in the gene body (Zhang et al., 2009; Xiao et al., 2016), and H3K4me2/3 in the gene body might contribute to the transcriptional memory in *Arabidopsis* (Alvarez-Venegas and Avramova, 2005; Ding et al., 2012; Kim et al., 2012). Moreover, when the presence of epigenetic tags was analyzed in different stress gene regions, the tendency of histone changes was similar or even less marked in the promoter rather than in the coding region of the gene (e.g., Shen et al., 2014; Crespo-Salvador et al., 2018).

## Materials and Methods

### Plant Material

Sterile potato plant *Solanum tuberosum* L. cultivar “Sarpo Mira” (carrying the *R* genes: *R3a*, *R3b*, *R4*, *Rpi-Smira1*, and *Rpi-Smira2*), obtained from the Potato Genebank (Plant Breeding and Acclimatization Institute – IHAR-PIB in Bonin) was initially derived from an *in vitro* tissue culture and kept in sterile soil in a phytochamber with 16 h of light (180 μmol m^-2^ s^-1^) at 18 ± 2°C and 60% humidity up to the stage of 10 leaves. Vegetative and generative descendants derived from tubers and seeds (F_1_) were obtained from unprimed and primed parental plants (F_0_), respectively. The F_1_ plants were cultivated and inoculated under the same conditions as F_0_.

### Pathogen Culture

*Phytophthora infestans* (Mont.) de Bary, virulent for “Sarpo Mira” (A1 mating type, race 1.2.3.4.6.7.10., isolate MP 977), was kindly supplied by the Plant Breeding and Acclimatization Institute (IHAR), Młochów Research Centre, Poland. The pathogen was grown on a cereal–potato medium and transferred two times through the potato tuber before infection.

### Immunization With BABA Treatment and Challenged Inoculation

The potato cv. “Sarpo Mira” was immunized by spraying potato leaves with 5 mM of BABA (3 ml per plant). BABA was delivered to the plant surface using an atomizer. The control of non-induced plants was sprayed with water (Floryszak-Wieczorek et al., 2015). At 72 h after immunization with BABA treatment, potato plants were challenge inoculated by spraying leaves with 5 ml of the oomycete zoospore suspension at a concentration of 2.5 × 10^5^ per 1 ml of water. For the purpose of disease assessment inoculated plants were first kept for 12 h at 100% humidity and 18°C. Next plants were moved to a growth chamber and they were kept under controlled conditions. The probes were collected at 0, 1, 3, 6, 24, 48, and 72 h after BABA treatment and 1, 3, 6, 24, and 48 h after *P. infestans* challenge inoculation. Progeny of unprimed and primed plants were challenge inoculated by *P. infestans* and probes were collected at 1, 3, 6, 24, and 48 hpi.

### Assessment of Disease Index

The area affected by disease symptoms was assessed on potato leaves 5 day after inoculation with *P. infestans* based on the scale of area under disease progress (AUDP) from I to IV (James, 1971), which represented the percentage of leaf area covered by late blight symptoms (I = 1–9%; II = 10–24%; III = 25–49%; IV = 50–100%). Disease symptoms were also determined during trypan blue staining of the *P. infestans* hyphae according to the assay proposed by Wilson and Coffey (1980). The AUDP was measured at parental F_0_ and F_1_ progeny plant using the open source ImageJ software.

### Gene Expression Analysis

The RNA was extracted from 200 mg of frozen leaf tissues using TriReagent (Sigma, United States). The obtained RNA was purified with the use of a Deoxyribonuclease Kit (Sigma, United States). For the reverse transcription 1 μg of RNA from every experimental variant was processed with a Reverse Transcription Kit (Thermo Scientific Fermentas, United States). Real-time PCR was performed on a Piko Real Thermocycler (Thermo Fisher Scientific, United States). Primers for the investigated genes (*CAF-1*, *H3*, *H4*, *HDAC*, *HAT*, *SUVH4*, *JMJ706*, *TrxG*, *NPR1*, *PR1*, *PR2*, and *SNI1*) were designed by the Primer3 Output software or the PRIMER BLAST (**Supplementary Table S1**). All of them were based on available potato (or *Solanaceae*) cDNA sequences found in the NCBI (GenBank) or PGSC (Potato Genome Sequencing Consortium). The reaction mixture contained 0.1 μM of each primer, 1 μl of 5× diluted cDNA, 10 μl of the Power SYBR Green PCR Master mix (Applied Biosystems) and DEPC treated water to the total volume of 20 μl. Reaction specificity was confirmed by the occurrence of one peak in the melting curve analysis. The data were normalized to the reference genes encoding the elongation factor (*ef1*α, AB061263) and *18S rRNA* (X67238). The Ct values were determined with the use of a Real-time PCR Miner (Zhao and Fernald, 2005) and the relative gene expression was calculated with the use of the efficiency corrected calculation models presented in Pfaffl (Pfaffl, 2001; Tichopad et al., 2004).

### Chromatin Immunoprecipitation Assay

ChIP was performed as previously described by Haring et al. (2007). Potato leaves (2 g) were fixed to crosslink protein–DNA interactions in a buffer with 1% formaldehyde and frozen at -80°C. One day before chromatin extraction, agarose A beads (Merck Millipore) were blocked and resuspended in the ChIP dilution buffer with the following specific antibodies: H3K4me2 (EMD Millipore; cat.-no 04-790), H3K9me2 (EMD Millipore; cat.-no. 07-441), or H3K27me3 (EMD Millipore; cat.-no. 07-449), and incubated overnight on a rotating wheel at 4°C to promote antibody attachment to the beads. Chromatin was isolated according to the protocol (Haring et al., 2007; Komar et al., 2016) and sheared by sonication. The resolution obtained by the ChIP procedure was determined by the size of the chromatin fragments used as input material. Ideally, the bulk of chromatin was sonicated to a length between 250 and 750 bp. Any insoluble materials were precipitated by the preclearing step, followed by overnight probe incubation with the antibody. Simultaneously “Input” probes (sonicated chromatin without the antibody) were precleared at blocked protein Agarose A beads. The next step consisted of chromatin reverse crosslinking. Then probes were reverse crosslinked by overnight incubation with NaCl and 20% SDS at 65°C with shaking. The next step was adding proteinase K to digest proteins, release and clean DNA. Samples were purified using a commercial spin column kit (Promega). The final step consisted of measuring the abundance of binding sites in the immunoprecipitated DNA by qPCR. The reaction mixture contained 0.1 μM of each primer, 2–5 μl purified DNA, 10 μl of the Power SYBR Green PCR Master mix (Applied Biosystems) and DEPC treated water to the total volume of 25 μl. Reaction specificity was confirmed by the occurrence of one peak in the melting curve analysis. Primers for the investigated genes (*NPR1*, *PR1*, *PR2*, *SNI*, and *WRKY1*) were designed by the Primer3 Output software. Data were analyzed by the “% of input method” (Komar et al., 2016). Raw Ct values were obtained after real-time qPCR reactions and adapted for input samples by subtracting a value of logarithm base 2 from the fraction of the input. The percentage of the input was calculated by applying the following formula: %input = 100^∗^2^[adjustedinput-Ct(sample)]^.

### Statistical Analysis

All the results were based on three independent experiments. For each experiment, at least three biologically replicated samples were collected, each consisting of leaves from six plants. For each experiment, means of the obtained values were calculated along with standard deviations. The analysis of variance was conducted and the least significant differences (LSDs) between means were determined using Tukey’s test at the level of significance α = 0.005.

## Results

### BABA-Primed Intergenerational Resistance to *Phytophthora infestans*

**FIGURE 1:**
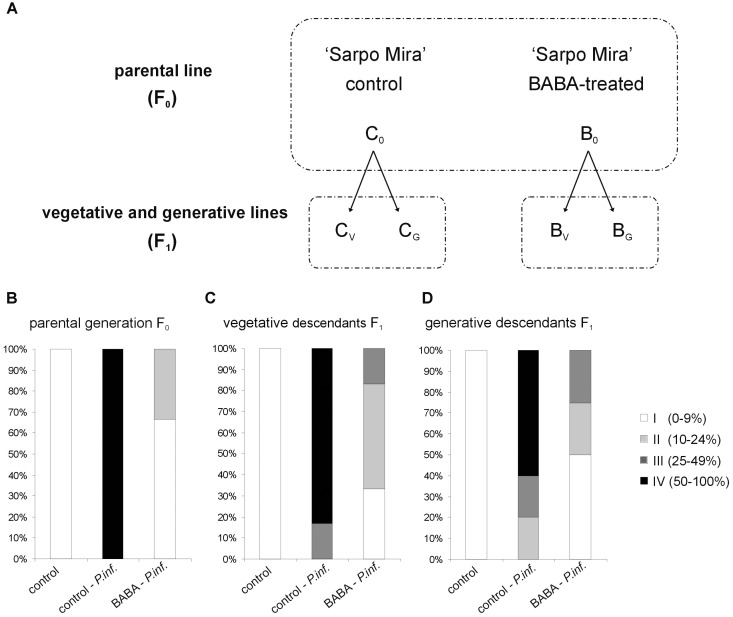
Intergenerational resistance in potato progeny unprimed and BABA-primed to *P. infestans*. Schematic representation of the experimental design **(A)**; the parental line of “Sarpo Mira” potato genotype (F_0_) was treated once with 5 mM BABA (B_0_). The progeny (F_1_) grown from tubers or seeds were not again treated with BABA, but only inoculated with *P. infestans* - at the stage of 10 compound leaves. Abbreviations mean: the parental F_0_ line BABA-primed – B_0_, the offspring of primed plants of the vegetative F_1_ line – B_V,_ and the generative F_1_ line – B_G_. Three days after BABA-exposure or water treatment (C_0_) potato leaves were challenged with *P. infestans* and disease development was assayed at day 5 after *P. infestans* inoculation (dpi). BABA-induced resistance against *P. infestans* in the parental (F_0_) line **(B)**, intergenerational resistance in the vegetative F_1_ progeny **(C)**, and in the generative F_1_ progeny **(D)**. Disease index is based on a 1–4 point scale, which represents the percentage of leaf area covered by late blight symptoms. Values represent means of at least three independent experiments, each with at least three biological replicates. Asterisks indicate values that differ significantly from unprimed (water treated) or unprimed and *P. infestans* inoculated potato leaves at *P* < 0.05 (^∗^), respectively.

Priming efficiency of 5 mM BABA in triggering SAR responses in “Sarpo Mira” was determined on the basis of the disease index assay, i.e., the development of potato late blight symptoms in the parental line (F_0_) and its successive progenies (F_1_), according to the experimental model shown in **Figure 1A**. Three days after BABA treatment (72 h) plants were inoculated and the rate of potato leaves colonized by *P. infestans* was scored at day 5 after the challenge. All the observed changes were referred to the BABA-unprimed plants, i.e., potato not subjected to priming or only inoculated with a virulent pathogen. The index of disease development in potato leaves represents the percentage of leaf area covered by late blight symptoms, respectively, classifying them into four categories according to the percentage of leaf tissue colonized by the pathogen. In the parental line (F_0_) supplied with BABA the late blight disease limitation was very high, resulting in approx. 70% disease spot reduction, compared to the unprimed one (**Figure 1B**). Less than 10% of potato leaf area was occupied by non-spreading late blight spots.

To determine the duration of priming state, the vegetative progeny of primed plants derived from tubers and generative progeny from seeds were analyzed. However, it should be underlined that the parental line (F_0_) was treated once with 5 mM BABA. This means that its progeny plants (F_1_) grown from tubers or seeds were not again treated with BABA, but only inoculated with *P. infestans* at the stage of 10 compound leaves. Our results showed that the BABA-primed state for effective defense of “Sarpo Mira” was transmitted from parents to their vegetative and generative progenies as enhanced resistance to the pathogen. Plants (F_1_) grown from tubers previously activated by BABA showed an important reduction of late blight symptoms (**Figure 1C**). Inoculated potato leaves from F_1_ plants had on average significantly smaller lesion diameters (up to 9% and 24% of the leaf area) than those from the unprimed and *P. infestans* inoculated ones.

Similarly, a considerable decrease (approx. 50%) of disease spot area compared to the infected unprimed leaves was found in the generative descendants not exposed to an additional stimulus (**Figure 1D**). These data showed that the offspring of BABA-primed potato, subjected to the same-generation priming, over one stress-free generation maintained an enhanced resistance to *P. infestans*.

### Transcriptional Reprogramming of SA-Dependent Genes After BABA Treatment

**FIGURE 2:**
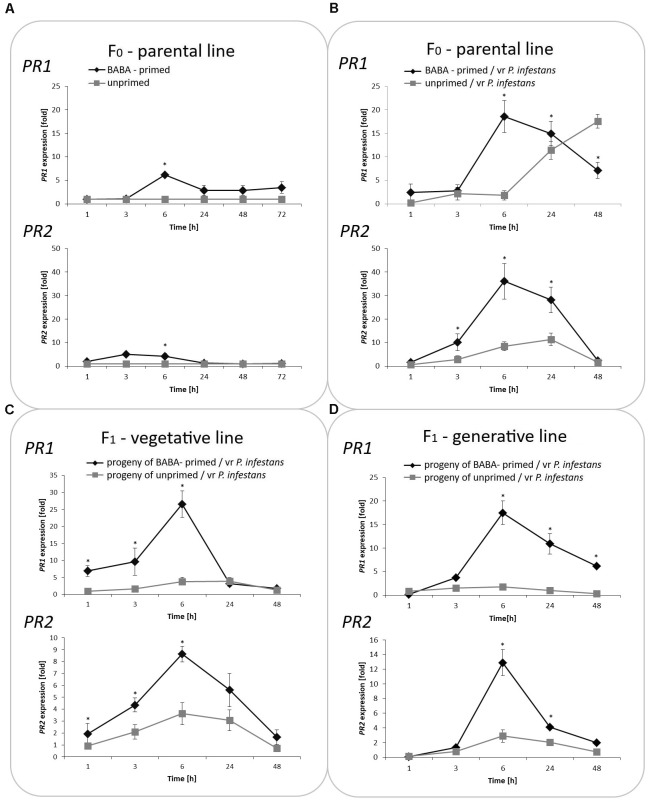
Treatment with BABA reprograms SA-dependent gene transcription. The RT-qPCR analysis of *PR1* and *PR2* gene expression in BABA primed leaves of potato plants followed by challenge inoculation with *P. infestans* (at 72 h after BABA treatment) in parental line F_0_ **(A,B)**. Analyses were performed at 0–48 h after 5 mM BABA exposure and 1–48 hpi after challenge inoculation. Transcriptional priming of *PR1* and *PR2* gene expression after *P. infestans* inoculation in the offspring of BABA-primed F_1_- vegetative line **(C)**, and F_1_- generative line **(D)**. Light columns refer to unprimed, while dark columns - to primed plant progeny. Values represent means of data ± SD of at least three independent experiments. Asterisks indicate values that differ significantly from unprimed (water treated) or unprimed and *P. infestans* inoculated potato leaves at *P* < 0.05 (^∗^), respectively.

Generally, *PR*s transcript levels were very low or undetectable in potato plants not exposed to BABA. In turn, BABA pretreatment resulted in a slight and transient up-regulation of the mRNA transcript for *PR1* and *PR2*, which returned after 48 h to nearly basal levels (**Figure 2A**). In contrast, the sequential treatment of potato plants with BABA followed by *P. infestans* provided data on stress imprint activation, facilitating the acquisition of a competence to react faster and stronger after challenge inoculation, in the form of a potentiated rise in *PR1* and *PR2* levels of the gene expression upon pathogen treatment. An enhanced *PR* transcript accumulation was reflected in the increase of basal resistance to the late blight disease compared to unprimed, but inoculated potato.

Moreover, our data revealed that the priming stimulus was transmitted into the next generation in the form of intergenerational stress memory. Since descendants of the primed potato were derived from tubers or seeds, they showed a faster and higher transcription of *PR1* and *PR2* correlated with an enhanced intergenerational resistance to *P. infestans* in comparison to the inoculated progeny of unprimed plants (**Figures 2B,C**).

### Priming for Defense Is Supported by Enhanced Expression of both *H3*, *H4*, and *CAF-1* Histone Chaperon Genes in F_0_ Potato Progeny

To assess whether the effective priming observed in the BABA-treated potato and its progeny was due to an epigenetic regulation for the heritable defense we focused on exploring BABA-primed parental changes of histone modifications, which might be potentially associated with reprogramming of gene expression toward switching on and retention of information on previous stress exposure.

**FIGURE 3:**
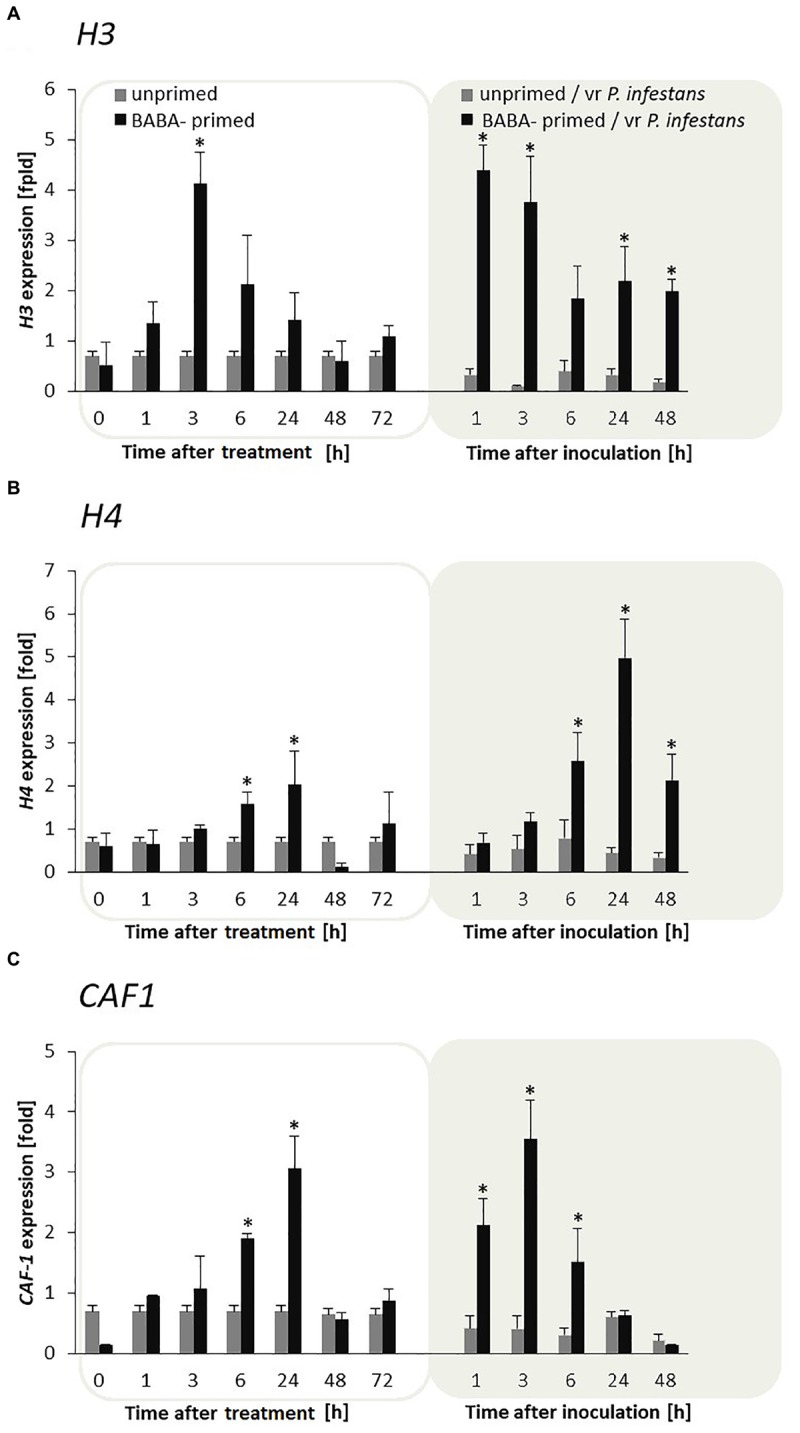
Priming for defense is supported by enhanced expression of both H3, H4, and CAF-1 histone chaperon genes in F_0_ potato progeny. The qRT-PCR analysis of *H3* **(A)**, *H4* **(B)**, and *CAF1* **(C)** gene expression in BABA-primed leaves of potato plants followed by challenge inoculation with *P. infestans* (at 72 h after BABA treatment). Analyses were performed at 0–48 h after 5 mM BABA exposure (white background) and 1–48 hpi after challenge inoculation (gray background). Light columns refer to unprimed, while dark columns – to primed plants. Values represent means of data ± SD of at least three independent experiments. Asterisks indicate values that differ significantly from unprimed (water treated) or unprimed and *P. infestans* inoculated potato leaves at *P* < 0.05 (^∗^), respectively.

Potato leaves pretreated with BABA showed sensitization to the *H3* and *H4* genes during the maintenance of the priming phase, and thus generated an enhanced expression of these genes, starting from 1 to 3 h in *H3* and from 1 to 24 h in *H4* after induction, respectively (**Figures 3A,B**). Moreover, the mRNA transcripts for both *H3* and *H4* were again early up-regulated during the first day after inoculation and afterward systematically diminished in the following time-periods after challenge inoculation compared to unprimed plants undergoing inoculation. Interestingly, the *CAF-1* gene of the histone chaperon protein revealed a clear correlation with the biphasic changes in *H3* and *H4* gene expression upon BABA and pathogen stress exposure in F_0_ potato plants (**Figure 3C**).

Moreover, *CAF-1* transcription levels were also much higher after inoculation with *P. infestans* in F_1_ offspring plants derived from tubers or seeds compared with the inoculated progeny of unprimed plants (**Supplementary Figures S1A,B**).

### Involvement of Histone Acetylation and Deacetylation in BABA-Primed Defense Information

**FIGURE 4:**
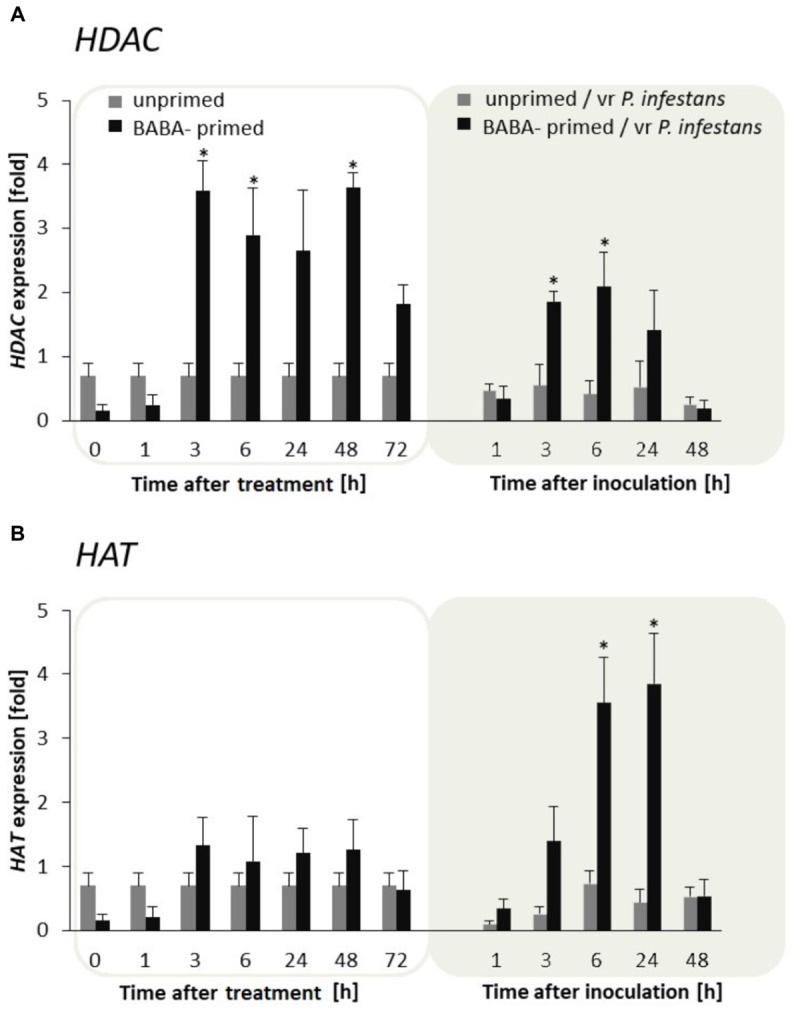
Expression patterns of histone acetylation and deacetylation upon exposure to BABA and biotic stress. Transcriptional analysis of *HDAC* **(A)** and *HAT* **(B)** gene expression in BABA-primed leaves of potato plants followed by challenge inoculation with *P. infestans* (at 72 h after BABA treatment). Analyses were performed at 0–48 h after 5 mM BABA exposure (white background) and 1–48 hpi after challenge inoculation (gray background). Light columns refer to unprimed, while dark columns - to primed plants. Values represent means of data ± SD of at least three independent experiments. Asterisks indicate values that differ significantly from unprimed (water treated) or unprimed and P. infestans inoculated potato leaves at *P* < 0.05 (^∗^), respectively.

To examine the flexibility of histone lysine acetylation in primed potato we analyzed gene expression of *HAT* and *HDAC* during the establishment of the priming and after triggering stress in the F_0_ potato line. An antagonistic tendency of *HAT* and *HDAC* transcriptional activities was observed (**Figures 4A,B**). Thus in BABA-treated potato leaves the relatively low *HAT* transcription levels changed after the challenge by the virulent pathogen when *HAT* gene expression gradually increased in successive hours after inoculation. In contrast, BABA treatment resulted in enhanced transcriptional priming of *HDAC* gene activity before inoculation preceding downregulation of this gene upon challenge with the oomycete pathogen.

In conclusion, the H3 acetylation seemed to be non-decisive for maintaining BABA-primed defense imprint, whereas the situation changed in plants triggered with pathogen when rapid and boosted *PR*s gene expressions might need HAT activity. In contrast to histone deacetylase, *HAT* transcription was also significantly intensified after challenge inoculation with *P. infestans* in F_1_ progeny plants (**Supplementary Figures S2A,B**). It is worth emphasizing that BABA triggered *HAT* expression only upon pathogen inoculation both in F_0_ and F_1_ progeny. Here, we found that the primed state in potato was tuned with the transcriptional memory of post-infection HAT activation in the next generation without an additional BABA treatment.

### Histone Lysine Methyltransferases and Demethylase Are Required for BABA-Triggered Immunity

**FIGURE 5:**
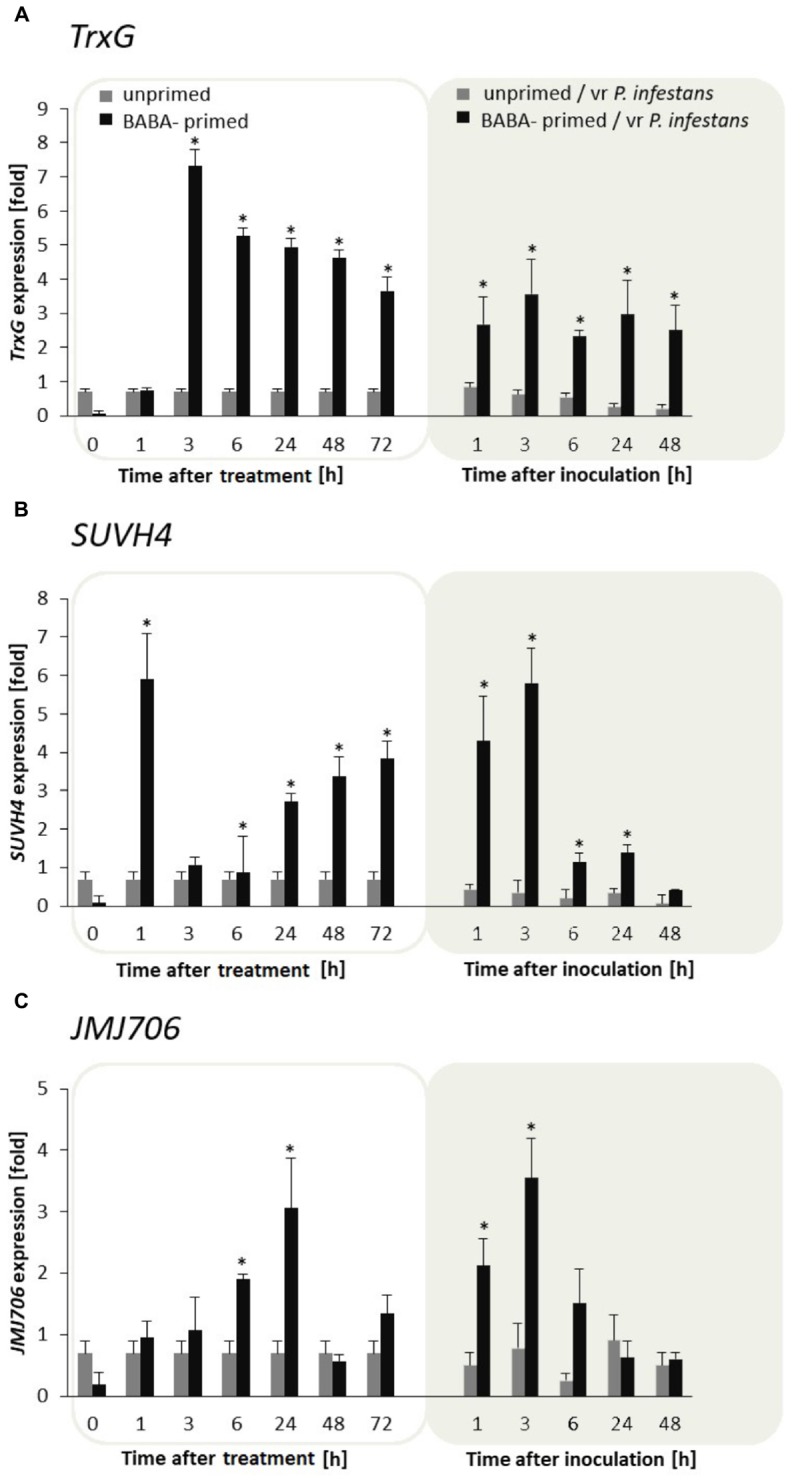
Histone lysine methyltransferases and demethylase are required for BABA-triggered immunity. Transcription changes in *TrxG*, *SUVH4*, and *JMJ706* gene expression in BABA-primed leaves of F_0_ potato plants followed by challenge inoculation with *P. infestans* **(A)**. Analyses were performed at 1–48 h after 5 mM BABA exposure (white background) and 1–48 hpi after challenge inoculation (gray background). Transcriptional priming of *TrxG*, *SUVH4*, *and JMJ706* gene expression after *P. infestans* inoculation in the offspring of BABA-primed F_1_- vegetative line **(B)**, and F_1_- generative line **(C)**. Light columns refer to unprimed, while dark columns - to primed plants progeny. Values represent means of data ± SD of at least three independent experiments. Asterisks indicate values that differ significantly from unprimed (water treated) or unprimed and *P. infestans* inoculated potato leaves at *P* < 0.05 (^∗^), respectively.

BABA turned out to be an effective factor in upregulation of the *TrxG* gene in potato leaves. The *TrxG* gene was first strongly (approx. 7-fold) potentiated at 3 h, and then the activity diminished slightly in the following hours after the BABA treatment and challenge inoculation. Nevertheless, generally *TrxG* transcript accumulation was much more abundant in induced rather than the unprimed and/or inoculated leaves (**Figure 5A**). An independent analysis of the *SUVH4* time expression pattern revealed a rather opposite effect to that of *TrxG* in the form of rapid growth (peaking at 1 h) followed by a short decrease (from 3 to 6 h) and repeated increase in *SUVH4* gene expression at the successive time points after BABA had been supplied (**Figure 5B**). Further data showed that *TrxG* and *SUVH4* were not so differentially expressed upon pathogen inoculation, initially giving enhanced gene activation. However, in F_1_ descendants of the primed potato, both levels of *TrxG* and *SUVH4* transcripts were significantly higher than in unprimed ones upon the triggering stress (**Supplementary Figures S3A,B**, **S4A,B**).

In turn, JMJ706 belonging to the family of demethylases reverses H3K9me2, was induced mainly upon BABA application and revealed gene upregulation (up to 4-fold increase) starting from 6 to 24 h after the inducer treatment, while it again sharply increased early upon pathogen stress exposure (**Figure 5C**). Interestingly, a similar, but much more marked increase in *JMJ706* transcription was found after *P. infestans* inoculation in plants derived from primed parents (F1 line) produced both from tubers and seeds (**Supplementary Figures S3C**, **S4C**).

### Tight Balance Between Antagonistic Histone Marks on *NPR1* and *SNI1* Genes During the Priming Phase

Next, we attempted to analyze specific histone modifications in the form of methylation at the lysine residue 4, 9 and 27 on histone H3 (H3K4me2, H3K9me2, and H3K27me3) on the *NPR1* and *SNI1* genes coding positive and negative regulators of SAR.

**FIGURE 6:**
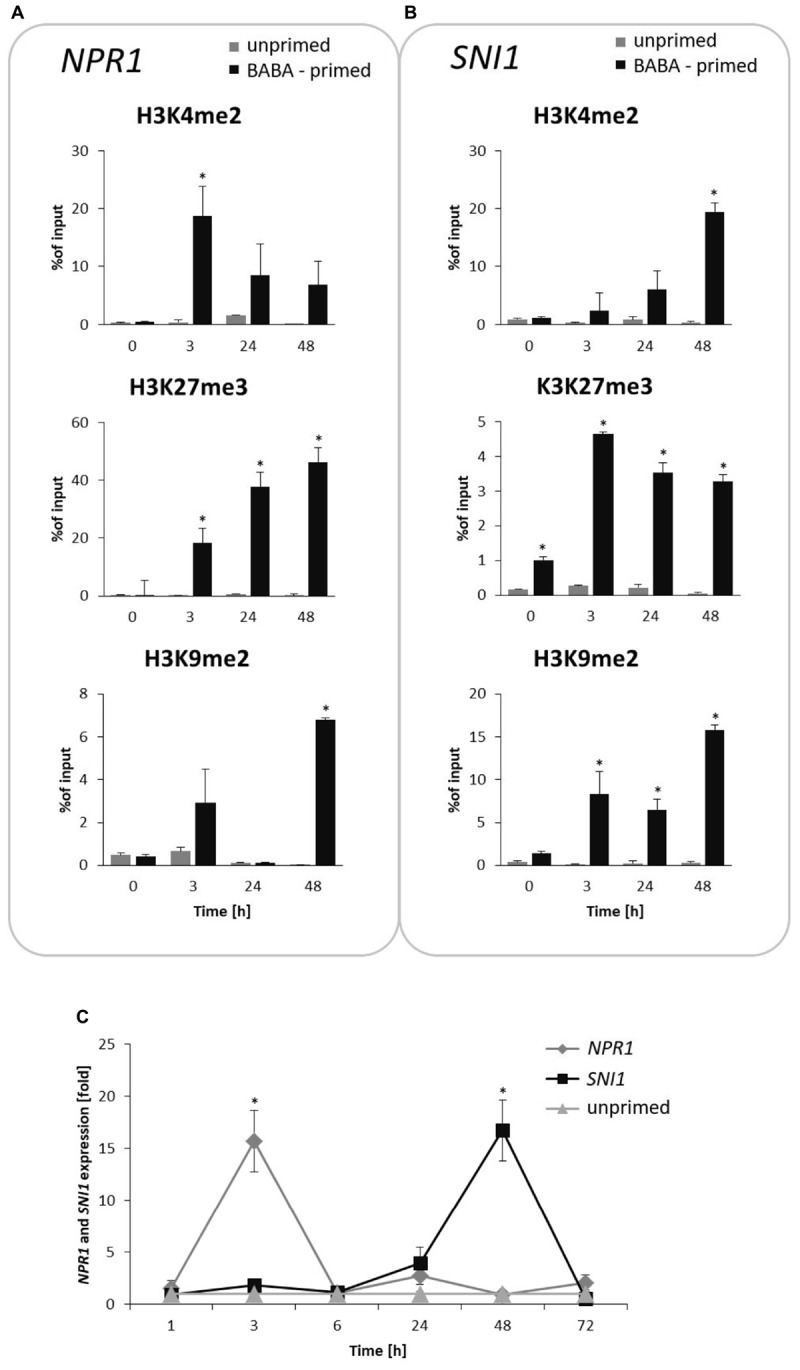
Switchable deposition of chromatin marks on the *NPR1* and *SNI1* gene, in which transcription antagonized silencing. Time-dependent H3 methylation profiles on *NPR1* **(A)** and *SNI1* **(B)** and their transcript levels **(C)** in the prime state before the triggering stress. ChIP-qPCR and RT-qPCR analysis of *NPR1* and *SNI1* gene expression were performed at 3, 24, 48, and 72 h after 5 mM BABA treatment. Each experiment was repeated three times and the data are presented as percentages of input DNA. Asterisks indicate values that differ significantly from unprimed (water treated) potato leaves at *P* < 0.05 (^∗^).

BABA induced an early (at 3 h) and significant increase in the H3K4me2 level on *NPR1*, associated with an enhanced gene transcription at the same time point after inducer treatment (**Figures 6A,C**). In turn, the H3K27me3 mark, initially being in reduced occupancy on *NPR1*, systematically increased (peaked at 48 h) after BABA supply and was tuned with downregulation of *NPR1* gene expression.

The time-dependent opposite tendency of histone methylation tags was found on the *SNI1* gene sequence (**Figures 6B,C**). The initially high accumulation of the H3K27me3 mark with a repressive level of *SNI1* transcription changed in the consecutive hours after induction and revealed abundant H3K4me2 occupancy under an enhanced *SNI1* gene expression (at 48–72 h). Interestingly, at the same hours, *NPR1* gene expression underwent downregulation. Results showed that epigenetic regulation of *NPR1*/*SNI1* was probably needed for tuning the transcriptional activity of SA-responsive genes for enhanced defense when establishing the priming phenomenon in potato.

BABA-exposure also resulted in high H3K9me2 levels on the *NPR1* gene loci appearing one time-point earlier (at 48 h) and preceding a strong increase (up to 15-fold) of this mark on the *SNI1* gene body at 72 h after induction (**Figures 6A,B**).

### Searching for the Epigenetic Mark on SA-Dependent Genes in Response to BABA-Priming

Then, we focused on *WRKY1* and two stress-responsive genes, *PR1* and *PR2*, with the transcriptional potential to store and maintain information on a previous stimulus for future use. As it was shown earlier (**Figure 2**), *PR1* and *PR2* as stress memory genes possess an ability, both in the parental and descendant lines, to produce significantly faster and higher levels of the transcript during the second triggering stress compared to the level in the first stimulus treatment. Previously it was found that *WRKY1* gene expression goes hand in hand with *PR1* transcription (paper under review). In order to find an epigenetic mark associated with transcriptional memory, we analyzed changes and durability of H3K4me2 and H3K9me2 occupancy on these genes during a priming phase and before the pathogen challenge in F_0_ and F_1_ lines of potato.

**FIGURE 7:**
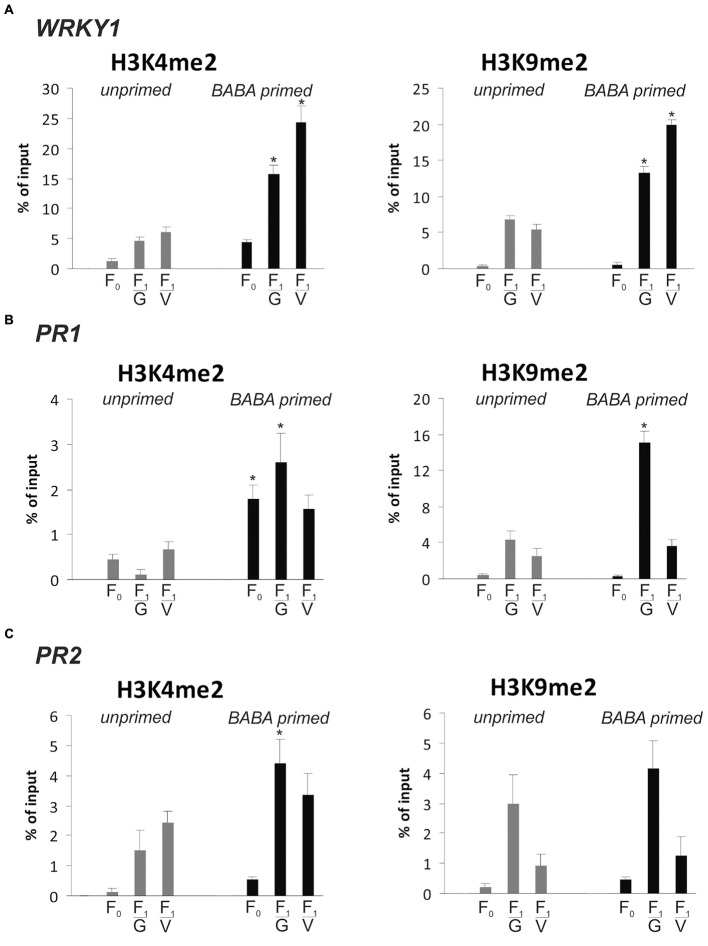
Epigenetic marks on SA-dependent genes in response to BABA-priming. Distribution levels of H3K4me2 and H3K9me3 on the gene body region of *WRKY1* **(A)**, *PR1* **(B)** and *PR2* **(C)** at the stage before the second challenge. ChIP-qPCR analyses were performed after 5 mM BABA (BABA-primed) or water treatment (unprimed), in the parental line (F_0_) and its descendants (F_1_) derived from tubers (F_1_/_V_) or seeds (F_1_/_G_). Each experiment was repeated three times and the data are presented as percentages of input DNA. Asterisks indicate values that differ significantly from unprimed (water treated) potato leaves at *P* < 0.05 (^∗^).

BABA pretreatment early induced a higher deposition of the H3K4me2 mark on the coding sequence of *WRKY1*, *PR1*, and *PR2*, compared to unprimed plants (**Figures 7A–C**). The elevated levels of H3K4me2 as the effect of the priming stimulus preceded a low and transient activation of *PR1* and *PR2* gene expression (**Figure 2A**). Importantly, before the pathogen stress enrichment of H3K4me2 occupancy was found on the gene body of *WRKY1*, *PR1*, and *PR2* in primed plants of the parental line (F_0_) and even more abundant in their descendants (F_1_) when the genes remained inactive or presented low transcription. Thus, the H3K4me2 tag might be regarded as a heritable mark for intergenerational regulation of resistance in potato.

Independently, we provided evidence that BABA application also modified the methylation status of H3K9me2 on the coding sequence of analyzed genes in parents and mostly in their offspring, which might also create a long-lasting post-stress memory in potato plants (**Figures 7A–C**).

## Discussion

Despite abundant reports on BABA-induced immune or adaptive defense responses against environmental challenges in the same plant generation, experimental research on BABA-primed stress imprint extending from one stressed plant generation to at least the first stress-free descendants has been scarce (Luna et al., 2012; Slaughter et al., 2012; Floryszak-Wieczorek et al., 2015; Martínez-Aguilar et al., 2016).

Our experiment clearly showed that the used potato genotype exposed to BABA displayed improved disease resistance and an enhanced capacity to mobilize faster and stronger defense responses to *P. infestans* within the same (F_0_) and in the next vegetative and generative progeny of primed plants (F_1_) when compared to unprimed parental plants and their progeny. It was observed that primed potato plants further changed their response pattern to regulate a network of SA-dependent gene expression differing from that involved in unprimed plants. The state of an intergenerational resistance to pathogen correlated with the transcriptional memory of gene expression, which we observed, encouraged us to investigate whether a transient plant exposure to BABA leads to chromatin modifications that could be maintained through mitotic or meiotic cell divisions and thereby preserve a particular expression pattern of stress response genes.

We identified specific histone H3 modifications that are known to be potentially associated with transcriptional reprogramming of gene expression toward the retention of information on previous stress exposure. BABA-primed potato exhibited the biphasic changes in gene expression for the *H3*, *H4*, and *chromatin assembly factor 1* (*CAF1*), being an H3-H4 histone chaperone. The first upregulation of these genes was found immediately after BABA treatment, while the other appeared upon the challenge inoculation.

Recently it was found in *Arabidopsis* leaves, as a non-dividing cell system, that the histone chaperone CAF-1 is required to establish a repressed chromatin state at defense genes. The *CAF-1* mutant (*faciata 2* mutant) defective in CAF-1 activity showed SA and BABA-induced activation of defense genes comparable to a constitutive priming response (Mozgová et al., 2015).

There is a good reason to believe that the biphasic changes in gene expression for *CAF1* observed by us in primed potato leaves might be associated with preventing overexpression of stress-responsive genes when the first stress was over. Nevertheless, more knowledge is needed on how CAF-1 deposits H3-H4 to provide a greater understanding of the mechanism for the maintenance and inheritance of histone modifications either through nucleosome recycling or copying of a proper modification into the newly incorporated histone (Mattiroli et al., 2017).

Based on the histone lysine acetylation results an antagonistic interaction between *HDA*s and *HAT*s gene expressions was observed in BABA-primed potato leaves. This outcome is in agreement with the proposal that both HATs and HDACs work in a global, untargeted fashion, and broadly affect the plant genome in two different manners (Shahbazian and Grunstein, 2007). Moreover, the obtained evidence showed that BABA did not activate *HAT*s until the plants were stimulated by the second stress stimulus. Thus histone acetylation increased mainly after the triggering stress and it was related to the augmented expression of the *PR1* and *PR2* genes.

Also, the finding of Luna et al. (2014) documented that acetylation was not essential for long-lasting BABA priming of SA-inducible genes within one *Arabidopsis* generation. Nevertheless, this does not preclude the supposition that H3K9 acetylation may perform an independent role in targeting potential genes in the priming state (Jaskiewicz et al., 2011; Luna et al., 2012). Generally, histone acetylation by HATs is associated with transcriptional activation, while histone deacetylation by HDACs is considered to be transcriptional inhibition tuned with the de-repression of SA-based defenses (Choi et al., 2012). Despite the above, HDA6 and HDA19 may be associated with transcriptional activation of the JA/ET-induced *PR*s genes (Zhou, 2005; Wu et al., 2008). Moreover, it is hypothesized that HDA6 is necessary for erasing histones, before RNA Polymerases (Pol IV) and methyltransferases may be involved in DNA methylation in order to provide locus-directed chromatin silencing (Liu et al., 2010; Blevins et al., 2014).

In our study, the transcript profiles of *HDAC* showed an enhanced expression upon BABA treatment followed by a decrease, but they maintained an elevated level under the triggering stress in comparison to unprimed-inoculated leaves. As it was hypothesized in *Arabidopsis*, HDA19 by modifying chromatin to a repressive state could prevent redundant over-activation of defense responses in the absence of the pathogen or, when needed, prepare an efficient expression without overestimation of defense responses under pathogen attack (Choi et al., 2012).

Next, among the other histone modifications, we focused on lysine methylation, being a more stable modification than acetylation, with the function in affecting gene expression as well (Morgunkova and Barlev, 2006). In order to assess whether histone H3 methylation mediated by methyltransferases TrxG or SUVH4 could play a role in the regulation of BABA priming response in potato, possible alternations between their transcript profiles were analyzed. The data revealed that the *TrxG* gene expression was early and strongly up-regulated upon BABA supply when compared to the unprimed potato. Interestingly, afterward the level of mRNA transcript accumulation for *TrxG* gradually diminished also after challenge inoculation, although TrxG transcription still remained higher when compared to unprimed-inoculated leaves.

Experimental evidence confirmed that TrxG proteins as chromatin regulators are involved in switching genes on and keeping them active (Schuettengruber et al., 2011, 2017). This could mean that the post-translational H3K4 di- and tri-methylation managed by the TrxG protein might serve as a molecular starter for a memory controlling subsequent priming response (Ding et al., 2012; Fromm and Avramova, 2014). In particular tri-methylation of lysine 4 on histone 3 (H3K4me3) is a hallmark of active genes in plant and animal systems; however, in plants di-methylation is also implicated in global gene activation (Lusser, 2002; Po-Wen et al., 2013).

As documented in other reports, *SUVH4* belonging to the PcG group represses thousands of stress-regulated genes by H3K27me3 or H3K9me2 for heritable heterochromatin assembly (Kleinmanns et al., 2017). We also found in our analyses that *SUVH4* presented an antagonistic tendency of transcription compared with the *TrxG* gene expression after BABA exposure.

The JMJ706 protein has been suggested to function as a histone demethylase and to specifically reverse di- and tri-methylation of H3K9, thus disassembling heterochromatin from the repressive state (Sun and Zhou, 2008; Qian et al., 2015). The presented data showed that *JMJ706* transcription increased immediately after the recognition of the BABA signal, while also its transcription levels were much higher after inoculation with *P. infestans* in the offspring plants (F_1_). These results correlated with an enhanced accumulation of H3K9me2 and H3K4me2 on the SA-dependent genes.

Interestingly, it was documented that H3K4me3 can also act as a docking site for H3K9me2 demethylases (Horton et al., 2010). Accumulated data revealed that epigenetic marks cannot be analyzed alone, but rather in combination with various histone modifications and their time-dependent mutual relationships define the open/close chromatin structure and create transcriptional competence to store information in the form of transcriptional memory of trainable genes (Avramova, 2015; de la Paz Sanchez et al., 2015; Pu and Sung, 2015; Fletcher, 2017).

Indeed, ChIP with RT-qPCR analyses in primed potato leaves revealed basic time-dependent differences in relation to the three analyzed histone methylation marks (H3K4me2, H3K27me3 and H3K9me2) on the gene body region of *NPR1* and *SNI1*, which encode opposite key regulators of plant immunity. Another finding of interest showed that BABA induced an epigenetic mechanism, in which the chromatin state switchable by transcriptional activation or repression of the *NPR1* and *SNI1* genes probably yielded input for reprogramming of the SA-dependent genes and contributed to the stress imprinting.

It is in line with other published results, indicating that mutually antagonistic bivalent histone marks, H3K4me2/3 and H3K27me3, have to be tightly balanced even at the same locus (Du et al., 2013; Trindade et al., 2017).

Our data indicate that H3K4me2/TrxG was probably required for the initial *NPR1*-dependent immune triggering, while H3K27me3/SUVH4 was needed rather for the establishment and maintenance of the histone methylation pattern, required to imprint the information for future use.

To date, despite many significant findings on the post-translational modifications of NPR1, summarized in a review paper by Pajerowska-Mukhtar et al. (2013), experimental data on the possibility of an NPR1 interaction with chromatin remodeling proteins and DNA methylation remain poorly understood. It was documented that the Elongator complex subunit2 (ELP2) exhibiting HAT activity is required to regulate both *NPR1* and its target defense genes in *Arabidopsis* (Wang et al., 2013).

In turn, SNI1 as a negative regulator of SAR was apparently needed to dampen the BABA primed signal, transduced by the *NPR1* into transient *PR-1* and *PR-2* gene expression and engaged in the time-dependent regulation of the transcriptional potential distribution to maintain a positive cost-balance after priming to a second distant triggering stress by *P. infestans*.

When looking for a clarification of the epigenetic mechanism, by which SNI1 modifies transcription, it was postulated that the transcriptional repression activity could be achieved both by histone modification or chromatin remodeling, with SNI1 possibly forming a scaffold to interact with the transcription modulator (Cowell, 1994; Gaston and Jayaraman, 2003; Mosher et al., 2006). However, since then apart from *SNI1* much more has been learned concerning the regulation of *SNC1* (Suppressor of NPR1, Constitutive) expression by chromatin remodeling complexes, including ATX7 and SNC1-mediated *R* gene immunity (Xia et al., 2013; Johnson et al., 2015) and other chromatin remodelers (Zou et al., 2017). Unfortunately, both SNC1 and SNI1 downregulating NPR1 are encoded by various gene structures and they impair host resistance through different molecular mechanisms (Maldonado et al., 2014).

In plants, their capacity to reprogram gene expression associated with the transcriptional memory of stress involves heritable histone modifications seen as epigenetic marks (Avramova, 2015). The increased presence of H3K4me2/3 or/and H3K9ac inversely to the transcript accumulation was found at memory genes in response to biotic stresses (Jaskiewicz et al., 2011; Ramírez et al., 2013; Martínez-Aguilar et al., 2016). We investigated an enhanced occupancy of H3K4me2 tag on the gene body of *WRKY1*, *PR1*, and *PR2* concomitant with transcript downregulation detected in leaves of primed potato and its descendants before the triggering stress. Nevertheless, how this epigenetic mark might promote the state of potato ability to mount an effective defense against a later virulent pathogen attack remains largely unanswered. An interesting hypothesis was postulated in mammalian research, suggesting that H3K4me3 antagonizes *de novo* DNA methylation at some genomic loci by blocking the Dnmt3 DNA methyltransferase (Otani et al., 2009).

The research carried out to identify the long-lasting memory revealed the importance of H3K9/SUVH4 methylation in keeping repressive modification at specific genomic sites related to priming to subsequent modulating effective immune responses at a minimal fitness cost (Martinez-Medina et al., 2016). In our experiment, the BABA priming induced a temporary increase in the H3K9me2 level on stress-responsive genes during transcriptionally inactive states before the second stress. Such changes in the deposition of histone marks, noted mainly in F_1_ line genes derived from BABA-primed plants, might work as the heritable storage of information after priming.

Other experimental evidence documented that H3K9 methyltransferase by the KYP protein (SUVH4/KRYPTONITE) was necessary for long-lasting BABA-induced resistance in *Arabidopsis* (Luna et al., 2014). Interestingly, it was stated that H3K9me2 is necessary for DNA methylation in the CpNpG sequence context mediated by CHROMOMETHYLASE 3 in *Arabidopsis* (Jackson et al., 2002; Saze and Kakutani, 2011; Du et al., 2012). A differentially methylated DNA region is usually much more stable compared to the dynamic histone changes and could be transgenerationally propagated in an intact form through mitosis and meiosis.

In order to find a functional link between various epigenetic modifications, simultaneously we examined the DNA methylation and demethylation status in potato plants contributing to BABA-primed changes associated with the transcriptional memory to improve resistance against *P. infestans* (paper under review). The same experimental design revealed that transcriptional priming of some SA-dependent genes (*NPR1*, *WRKY1*, and *PR1*) was not directly due to DNA methylation. Among other interesting issues, the vegetative and generative offspring of primed plants carrying a less methylated *R3a* promoter showed an earlier and higher transcription of *R3a*, correlated with an enhanced intergenerational resistance to *P. infestans*, when compared to the inoculated progeny of unprimed potato.

## Conclusion

The offspring of BABA-primed potato, subjected to the same-generation priming, maintained an enhanced resistance to *P. infestans* over one stress-free generation. The obtained data revealed that a time-dependent and opposite combination of different histone modifications on the *NPR1* and *SNI1* gene loci via histone methyltransferases (TrxG / SUVH4) and demethylase (JMJ706), seems to be essential for a successful fixation of the priming and intergenerational resistance to *P. infestans*. BABA-triggered switchable chromatin states with an opposite transcription on the *NPR1* and *SNI1* genes might reprogram stress response *PR1* and *PR2* genes and confer competence to store information in the form of transcriptional memory associated with the H3K4me2 epigenetic mark. It is proposed that the H3K9me2 methylation pattern may act in potato as part of such an immune sensory system closely linked to other epigenetic changes, such as DNA methylation. In turn, histone acetylation had not been essential for the establishment of the priming state until potato plants were challenged by the second stress, when a rapid and boosted *PR*s trainable gene expression probably required HAT activity both in F_0_ and F_1_ progeny.

## Author Contributions

JF-W, MA-J, and JD planned and designed the research. BM and DK performed the experiments, and collected and analyzed the data. JF-W wrote the manuscript.

## Conflict of Interest Statement

The authors declare that the research was conducted in the absence of any commercial or financial relationships that could be construed as a potential conflict of interest.

## Supplementary Material

FIGURE S1 Transcriptional activity of *CAF-1* in primed potato progeny derived from tubers **(A)** and seeds **(B)** after inoculation with *P. infestans.* Analyses were performed at 1–48 hpi after challenge inoculation. Light columns refer to unprimed while dark columns – to primed plants subjected to inoculation. Values represent means of data ± SD of at least three independent experiments. Asterisks indicate values that differ significantly from unprimed and *P. infestans* inoculated potato leaves at *P* < 0.05 (^∗^), respectively.

FIGURE S2 Transcription patters of histone acetylation (*HAT*) and deacetylation (*HDAC*) in primed potato progeny derived from tubers **(A)** and seeds **(B)** after inoculation with *P. infestans*. Analyses were performed at 1–48 hpi after challenge inoculation. Light columns refer to unprimed, while dark columns – to primed plants subjected to inoculation. Values represent means of data ± SD of at least three independent experiments. Asterisks indicate values that differ significantly from unprimed and *P. infestans* inoculated potato leaves at *P* < 0.05 (^∗^), respectively.

FIGURE S3 Transcript levels of genes modifying histone methylation – *TrxG* **(A)**, *SUVH4* **(B)**, and demethylation *JMJ706* **(C)** in primed potato progeny derived from tubers after inoculation with *P. infestans*. Analyses were performed at 1–48 hpi after challenge inoculation. Light columns refer to unprimed, while dark columns – to primed plants subjected to inoculation. Values represent means of data ± SD of at least three independent experiments. Asterisks indicate values that differ significantly from unprimed and *P. infestans* inoculated potato leaves at *P* < 0.05 (^∗^), respectively.

FIGURE S4 Transcript levels of genes modifying histone methylation – *TrxG* **(A)**, *SUVH4* **(B)**, and demethylation *JMJ706* **(C)** in primed potato progeny derived from seeds after inoculation with *P. infestans*. Analyses were performed at 1–48 hpi after challenge inoculation. Light columns refer to unprimed, while dark columns – to primed plants subjected to inoculation. Values represent means of data ± SD of at least three independent experiments. Asterisks indicate values that differ significantly from unprimed and *P. infestans* inoculated potato leaves at *P* < 0.05 (^∗^), respectively.

TABLE S1 List of designed primers.
