# Supplementary figures and images for: BABA-Primed Histone Modifications in Potato for Intergenerational Resistance to Phytophthora infestans
Source: Front Plant Sci. 2018 Aug 29;9:1228. doi: 10.3389/fpls.2018.01228 (PMC6135045; doi:10.3389/fpls.2018.01228)

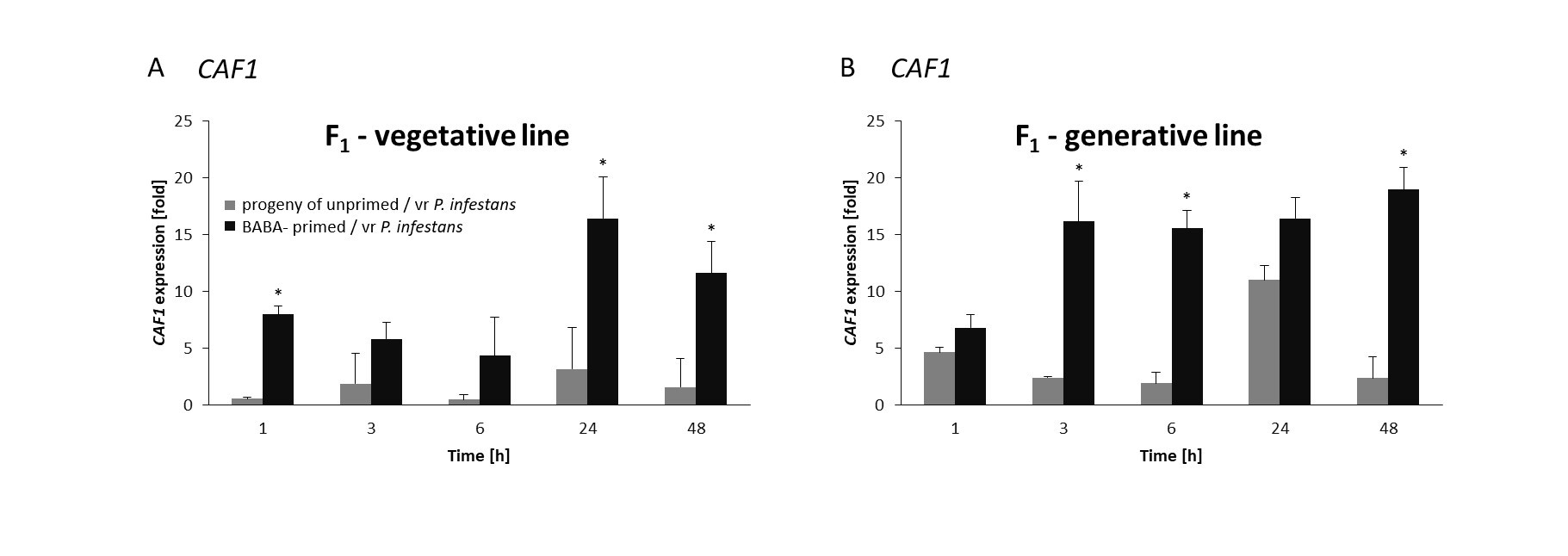

Supplement: FIGURE S1 — Transcriptional activity of CAF-1 in primed potato progeny derived from tubers (A) and seeds (B) after inoculation with P. infestans. Analyses were performed at 1–48 hpi after challenge inoculation. Light columns refer to unprimed while dark columns – to primed plants subjected to inoculation. Values represent means of data ± SD of at least three independent experiments. Asterisks indicate values that differ significantly from unprimed and P. infestans inoculated potato leaves at P < 0.05 (∗), respectively. [file Image_1.JPEG]

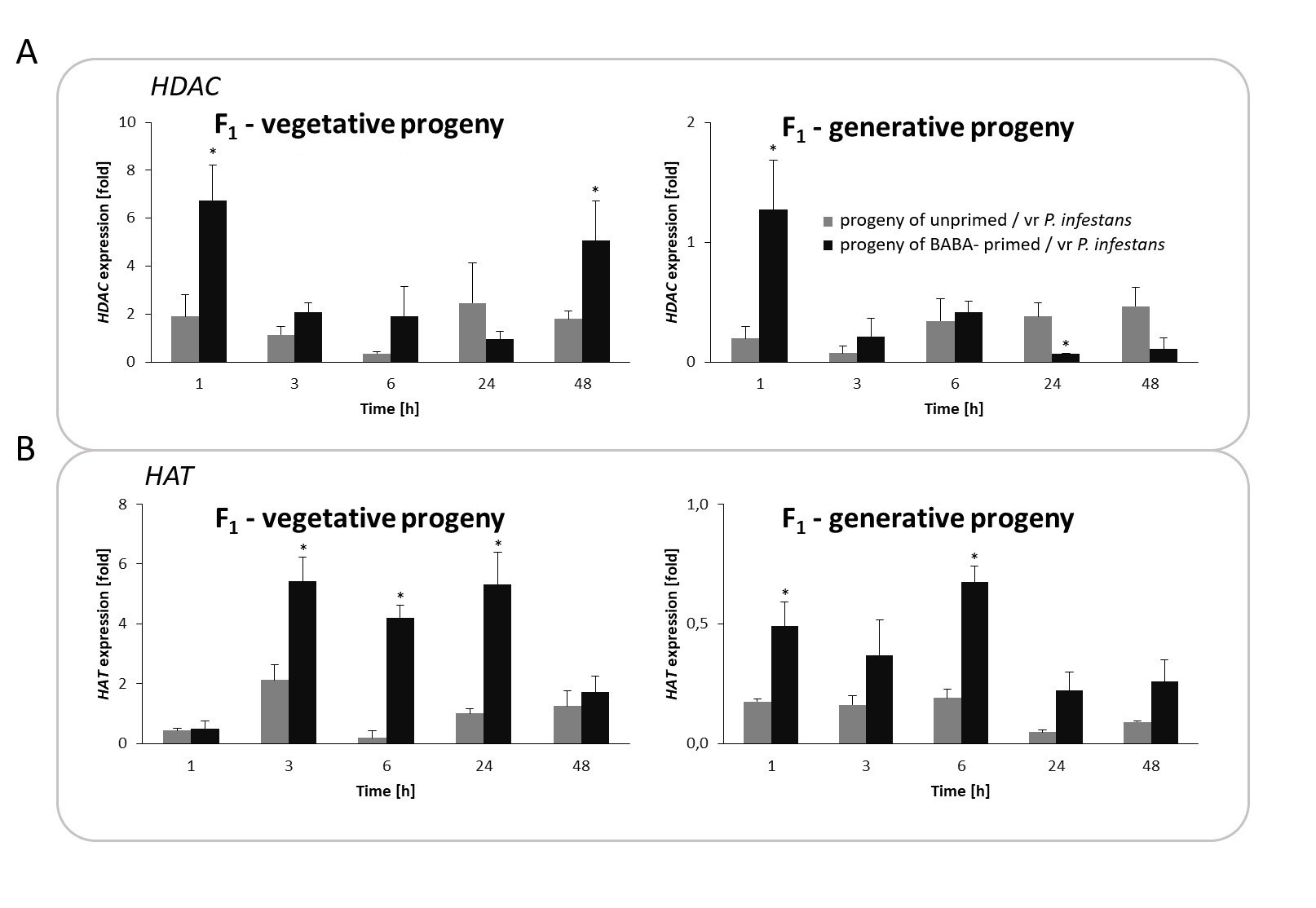

Supplement: FIGURE S2 — Transcription patters of histone acetylation (HAT) and deacetylation (HDAC) in primed potato progeny derived from tubers (A) and seeds (B) after inoculation with P. infestans. Analyses were performed at 1–48 hpi after challenge inoculation. Light columns refer to unprimed, while dark columns – to primed plants subjected to inoculation. Values represent means of data ± SD of at least three independent experiments. Asterisks indicate values that differ significantly from unprimed and P. infestans inoculated potato leaves at P < 0.05 (∗), respectively. [file Image_2.JPEG]

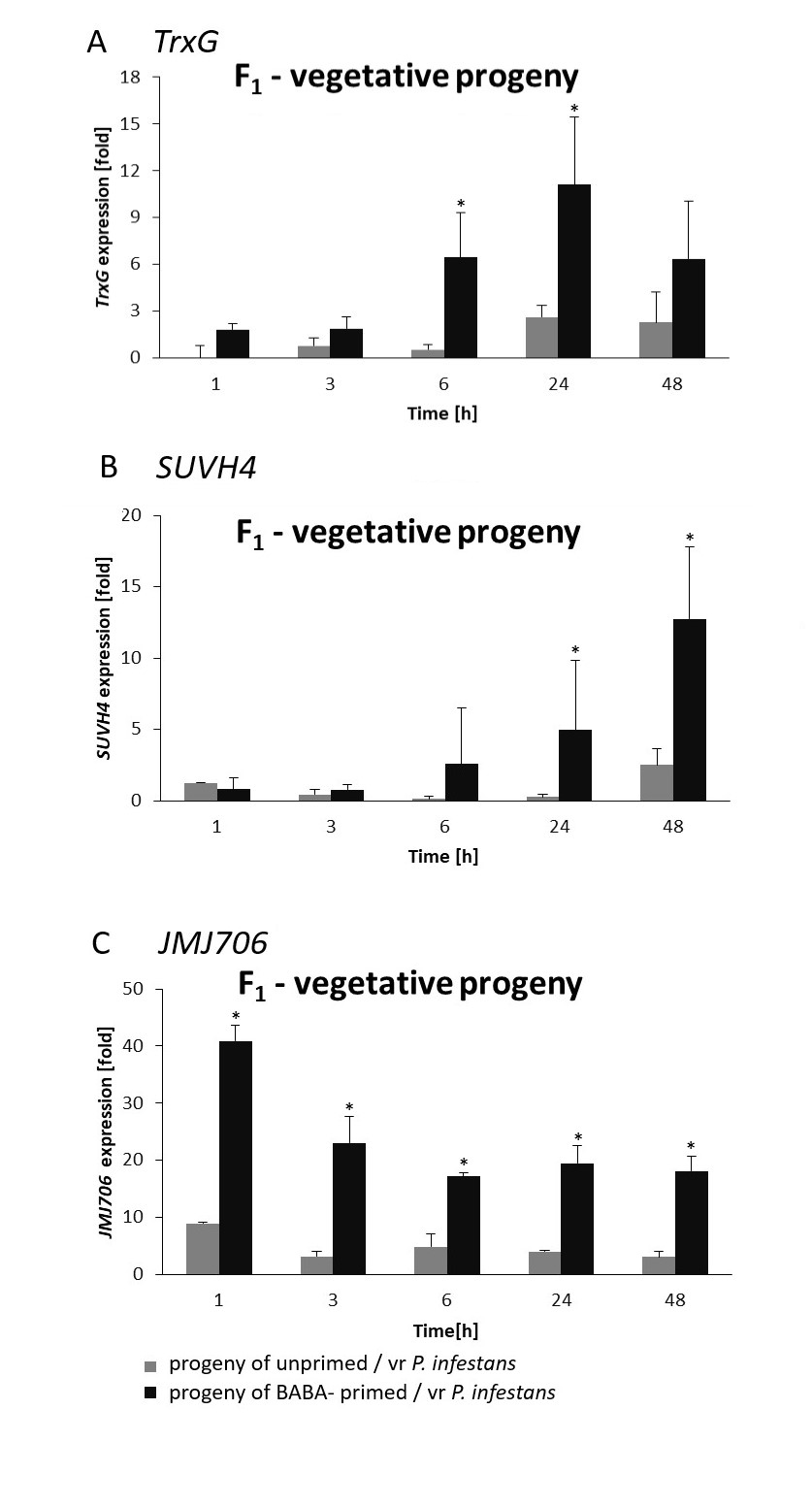

Supplement: FIGURE S3 — Transcript levels of genes modifying histone methylation – TrxG (A), SUVH4 (B), and demethylation JMJ706 (C) in primed potato progeny derived from tubers after inoculation with P. infestans. Analyses were performed at 1–48 hpi after challenge inoculation. Light columns refer to unprimed, while dark columns – to primed plants subjected to inoculation. Values represent means of data ± SD of at least three independent experiments. Asterisks indicate values that differ significantly from unprimed and P. infestans inoculated potato leaves at P < 0.05 (∗), respectively. [file Image_3.JPEG]

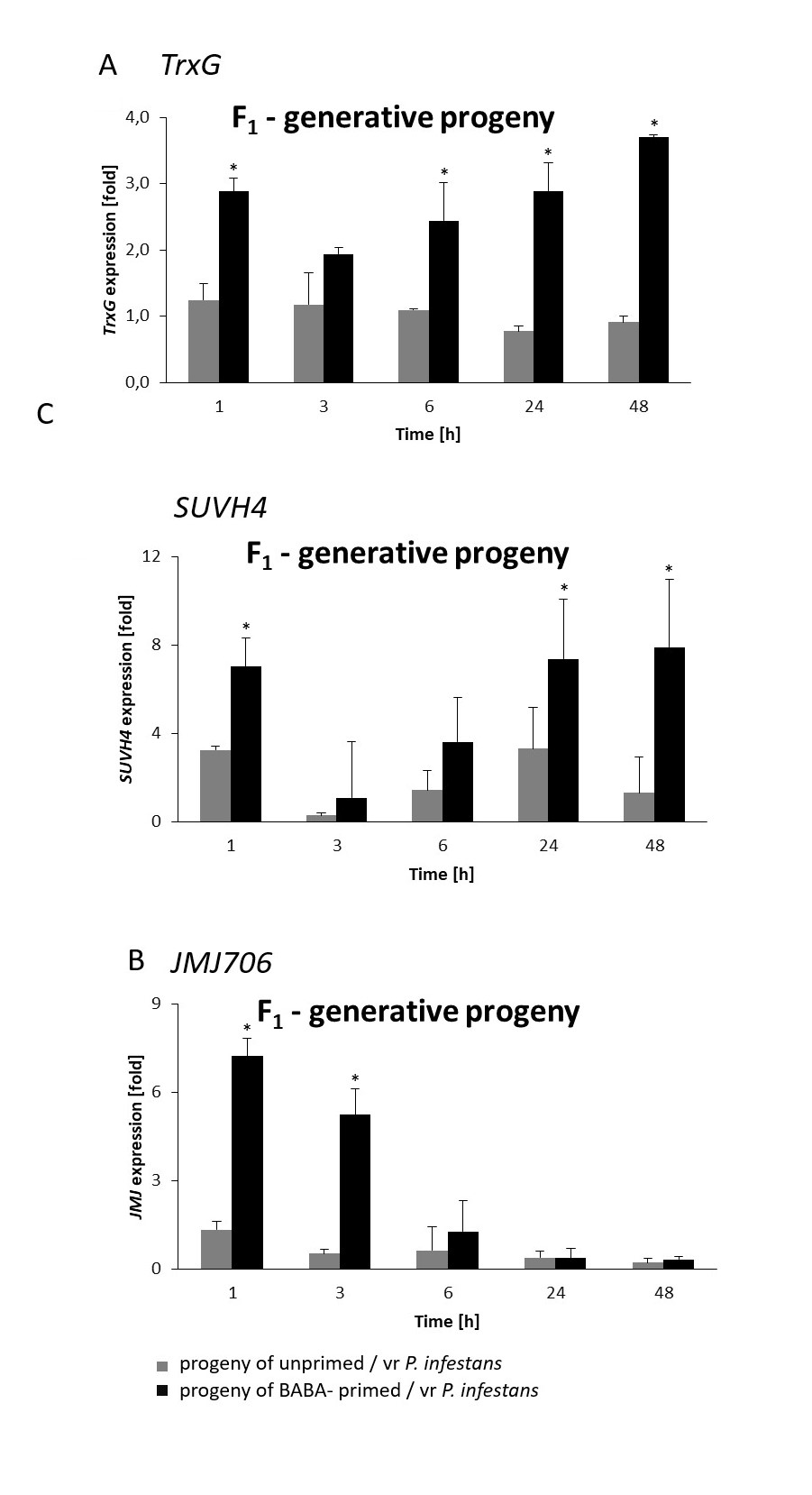

Supplement: FIGURE S4 — Transcript levels of genes modifying histone methylation – TrxG (A), SUVH4 (B), and demethylation JMJ706 (C) in primed potato progeny derived from seeds after inoculation with P. infestans. Analyses were performed at 1–48 hpi after challenge inoculation. Light columns refer to unprimed, while dark columns – to primed plants subjected to inoculation. Values represent means of data ± SD of at least three independent experiments. Asterisks indicate values that differ significantly from unprimed and P. infestans inoculated potato leaves at P < 0.05 (∗), respectively. [file Image_4.JPEG]
