# Supplementary material for: BABA-Primed Histone Modifications in Potato for Intergenerational Resistance to Phytophthora infestans
Source: Front Plant Sci. 2018 Aug 29;9:1228. doi: 10.3389/fpls.2018.01228 (PMC6135045; doi:10.3389/fpls.2018.01228)
Supplement: TABLE S1 — List of designed primers. [file Table_1.DOCX]

| gene | Forward | Reverse | Tm |
| --- | --- | --- | --- |
| *EF* | ATTGGAAACGGATATGCTCCA | TCCTTACCTGAACGCCTGTCA | 53°C |
| *CAF1* | CATGGCGGTCACAAAGCAAA | CTGCCACACTTGAAATGACCC | 53°C |
| *H3* | TTGCCCAGGACTTCAAGACT | GTCCTCAAAGAGACCCACCA | 52°C |
| *H4* | AAGAGGCAGGGAAGGACTCT | AAGAGGCAGGGAAGGACTCT | 53°C |
| *HDAC* | GTGTTCATGCTGGGCCCTAT | TCTTGGAACGTTGTGGCAGT | 52°C |
| *HAT* | ATGTGAATGGTGGTCGCTCT | GTCCAAGAGTTTGCAACAGCA | 53°C |
| *SUVH4* | TCCAAGAGTTTGTTCTTGTGCT | ACTGTAAGGCAACTTCCACCA | 53°C |
| *JMJ706* | TTATCAGCACTGTGGGGCAG | CACATGCTCTCGGACAACCT | 53°C |
| *TrxG* | CAACTTGCGTGTTATTGTGG | ATACAGCTTTGCCATTCGTT | 53°C |
| *NPR1* | GGTGCACCGATGCATTTTGT | AATAGGCGAGCACACTGACC | 53°C |
| *PR1* | GGGAGAAGCCAAACTACAACTATG | ACGAGCCCGACCACAACC | 54°C |
| *PR2* | GATGGAACGAACAGGAGGAG | GGCTTTCTCGGACTACCTTC | 53°C |
| *SNI-1* | CCAGAAAATGGAACACCGCC | AGCACACCCTAGGAAATCGC | 53°C |
| *18S rRNA* | GGGCATTCGTATTTCATAGTCAGAG | CGGTTCTTGATTAATGAAAACATCCT |  |
